# Selectivity Beyond Mass: Real‐Time Isomer Separation Using SLIM IMS Coupled to PTR‐MS

**DOI:** 10.1002/jms.70031

**Published:** 2026-01-28

**Authors:** Jacob Jordan, Alfons Jordan, Christian Lindinger, Gernot Hanel, Tobias Fügenschuh, Martin K. Beyer, Philipp Sulzer

**Affiliations:** ^1^ IONICON Analytik GmbH Innsbruck Austria; ^2^ Institut für Ionenphysik und Angewandte Physik Universität Innsbruck Innsbruck Austria

## Abstract

Proton‐transfer‐reaction—mass spectrometry (PTR‐MS) provides real‐time analysis of volatile organic compounds (VOCs) relevant to atmospheric research, food and flavor characterization, industrial process control, and medical applications. However, PTR‐MS is inherently limited in its ability to resolve isomers of molecules with the same mass‐to‐charge ratio (*m/z*). To address this selectivity constraint, we coupled a time‐of‐flight (TOF) based PTR‐MS setup with a “Structures for Lossless Ion Manipulations Ion Mobility Spectrometry” (SLIM IMS) module. The integration of SLIM IMS introduces an additional separation dimension without compromising the real‐time capability of PTR‐MS.

A custom designed SLIM device enabled efficient confinement and transmission of low mass ions (*m/z* < 300), which are typically analyzed in PTR‐MS. The developed system achieved helium‐based ion mobility resolutions (CCS_He_/ΔCCS_He_) of 190 for a path length of 9 m, corresponding to nitrogen‐based resolutions (CCS_N2_/ΔCCS_N2_) of approximately 600. On average 65% of the ions transmitted in straight‐through mode were still detected after accumulation and transport through the 9‐m serpentine IMS structure. Limits of detection were determined to be in the low parts‐per‐trillion by volume (pptv) range.

The enhanced resolving power of the PTR‐SLIM‐TOF was demonstrated by the successful separation of the protonated isomeric system 3‐methyl‐2‐butanone and 2‐pentanone (*m/z* 87.080) within multiple IMS laps. Furthermore, as a real‐life test, the headspace above freshly brewed coffee was analyzed. Separation of the isomeric flavor compounds, 2‐ethyl‐3‐methylpyrazine, 2,3,5‐trimethylpyrazine, and 2‐ethyl‐5(6)‐methylpyrazine was confirmed by introducing reference samples.

These results demonstrate that coupling PTR‐TOF‐MS with SLIM‐IMS substantially enhances compound selectivity while retaining the real‐time and sensitivity capabilities of PTR‐MS. The PTR‐SLIM‐TOF platform represents a significant advancement in ion mobility assisted mass spectrometry for high‐throughput chemical analysis across diverse scientific and industrial applications.

## 1 Introduction

Over the past few decades, developers of proton‐transfer‐reaction—mass spectrometry (PTR‐MS) instruments have pursued two key objectives: improving sensitivity and selectivity. PTR‐MS sensitivity is specified as the number of detected ion counts per second for 1 part‐per‐billion volume mixing ratio (cps/ppbv) of an analyte, typically in air. While the original developers of the technology reported 1 cps/ppbv in 1995 [1], only 3 years later the same group increased the sensitivity by over one order of magnitude to 10–20 cps/ppbv [2]. This upward trend has continued in subsequent years and can still be observed today, with “sensitivity milestones” being reported in 2009 with > 500 cps/ppbv [3], in 2012 with > 1000 cps/ppbv [4], in 2017/18 with up to 18 000 cps/ppbv [5, 6], and in 2023 with up to 80 000 cps/ppbv [7], to name just a few.

The developments in PTR‐MS selectivity were much less straightforward. First, we have to define selectivity issues, which have to be solved. In their 2019 report on advances in PTR‐MS, Pleil et al. [8] summarize the prevalent perception of the separation capabilities of PTR‐MS and similar direct injection mass spectrometry (DIMS) technologies, like SIFT‐MS. While prominent compound features in, for example, a humid air matrix can be tracked almost in real time through their molecular ions, the identification of trace compounds in complex mixtures is challenging because of the simultaneous presence of all ions. While high‐resolution PTR‐TOF‐MS instruments can now resolve isobars, that is, ions with the same integer *m/z* value, isomers featuring exactly the same *m/z* value cannot be distinguished.

The first attempt to solve all selectivity issues, separation of isobars and isomers, as well as unambiguous substance identification at once, was done by coupling conventional gas chromatography (GC(‐MS)) with PTR‐MS [9]. In a GC‐PTR‐MS device, the sample is first preseparated in a GC column before being analyzed by both methods: chemical ionization via PTR‐MS and standard electron ionization MS. This setup allows for unambiguous identification using well‐established GC–MS database related identification methods and the correlation to mass spectra obtained by PTR‐MS for each individual compound at its individual concentration. Cycle times of several tens of minutes and therefore the loss of the unique real‐time capability of PTR‐MS most probably is the reason why GC‐PTR‐MS never became widely used [10]. Multicapillary‐column [11] or fast‐GC [12] PTR‐MS setups perform at a somewhat reduced separation power compared to conventional GC but improve the time resolution to 1–3 min. This is still far from the sub‐second region of direct injection PTR‐MS, but a convenient trade‐off for specific fields of application, for example [10, 13, 14, 15].

Substantially greater success resulted from the increase in mass resolution, an enhancement initially triggered by the switch from quadrupole mass filters to TOF analyzers. Mass resolution is frequently defined as the position of a mass spectral peak's maximum “m” divided by the full width of this peak at half maximum height (FWHM) “∆m.” The first PTR‐TOF‐MS prototype introduced in 2004 by Blake et al. [16] performed at about 1500 m/∆m, which was “*not sufficient in most cases to resolve nominally isobaric species*.” Five years later, instruments with 6000 m/∆m became commercially available and allowed for clear separation of many common isobars, for example, ketene and propene, methylketene and butene, or furan and isoprene [17]. Nowadays, state‐of‐the‐art PTR‐TOF‐MS devices exceed 10 000–15 000 m/∆m [5, 6, 7], and it is foreseeable that this upwards trend in mass resolution will continue. However, assuming that high mass resolution is additionally paired with high mass accuracy, this only solves selectivity issues concerning isobar separation and identification. Isomeric compounds cannot be distinguished, even with ultimate mass resolution.

SIFT‐MS pioneered isomer separation. By switching reagent ions, characteristic product ions with different nominal *m/z* values can be produced during the ionization of isomers, for example, isomeric aldehydes and ketones [18]. This concept was adapted to PTR‐MS in the late 2000s [3]. Nowadays, reagent ion switching times of 1–2 s for changing between H_3_O^+^, NO^+^, O_2_^+^, and NH_4_^+^ are reported [7], that is, a process close to real‐time. Unfortunately, switching reagent ions is not a universal solution for isomer separation as the formation of unambiguous product ions is limited to certain compound classes or even particular compounds.

The bottom line of this brief historical overview is that, although there have been many successful approaches to increasing PTR‐MS selectivity, no method has yet addressed these contemporary analytical challenges, at least not without also entailing major disadvantages.

Ion mobility spectrometry (IMS) [19] is a well‐established rapid direct sample injection separation technique. Implementing the separation power of IMS into PTR‐TOF‐MS has the potential to finally resolve most selectivity issues without sacrificing real‐time capability. Among the various IMS embodiments, those based on travelling waves (TW; introduced by Giles et al. [20]) are especially beneficial, because the required voltage drop and the length of the ion mobility path are decoupled. Put simply, this means that IMS resolution can be increased more or less freely by increasing the ion mobility path length [19]. However, without any further measures, extending the ion mobility path would be expected to lead to a sensitivity diminishing loss of ions. As its name implies, the rather recent innovation “structures for lossless ion manipulations (SLIM)” (Pacific Northwest National Laboratory PNNL; Tolmachev et al. [21]) can drastically reduce such a loss of ions. Indeed, SLIM IMS‐MS devices, with their RF + DC ion confinement in a serpentine separation path, perform exceptionally well in terms of selectivity and sensitivity, and are commercially available since 2021 [22] (MOBILion Systems, PA, USA).

Enhancing the selectivity of chemical ionization (CI)–MS by implementing a SLIM IMS module between the CI and the MS part of an instrument has already been demonstrated. In their 2024 study, Chen et al. [23] investigated a wide range of alkylammonium ions using two different planar differential mobility analyzers coupled with TOF‐MS, while the relative mobility of five of the analytes was verified with a SLIM‐based CI‐IMS‐TOF utilizing benzene cations as reagent ions. Furthermore, there is a whitepaper (by the instrument manufacturer) evaluating the separation of methyl salicylate and methyl paraben in a SLIM IMS PTR‐TOF‐MS instrument with the PTR reaction chamber consisting of a resistive cylinder (DC voltage applied) surrounded by an RF‐only quadrupole [24].

Here, we present for the first time (to the best of our knowledge) the integration of a dedicated and optimized SLIM IMS module into a “classic” PTR‐MS instrument. With the term “classic,” we mean a design similar to the one introduced by the PTR‐MS inventors 30 years ago [1], that is, equipped with a hollow cathode reagent ion source and a PTR reaction chamber consisting of a series of metal ring electrodes separated by airtight isolating spacers and electrically connected via a resistor chain. This has the advantage that the ion chemistry is well defined and controlled, as documented in numerous publications, an ideal prerequisite for testing new instrumental developments.

## 2 Experimental Setup

**FIGURE 1:**
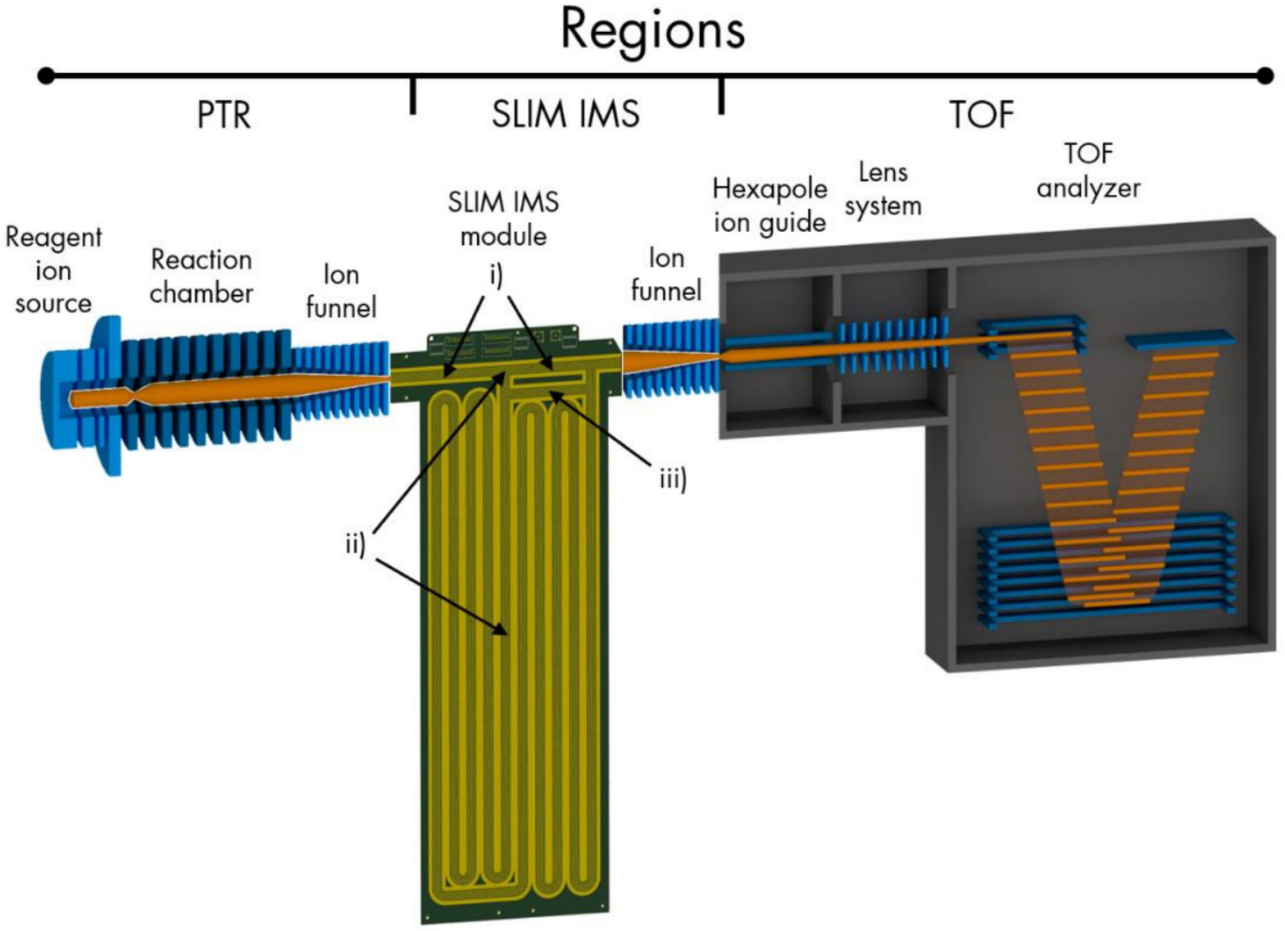
Schematic representation of the PTR‐SLIM‐TOF prototype. PTR region: reagent ion production and chemical ionization of the analytes. SLIM IMS region: either direct injection of ions into the TOF region or IMS separation, with (i) ion accumulation area/direct path, (ii) ion injection port followed by a serpentine SLIM IMS drift path, and (iii) loop path for multiple laps. TOF region: m/z separation and detection of the ions.

In Figure 1, a schematic view of the PTR‐SLIM‐TOF prototype is shown. To ensure clarity, the individual parts of the prototype are described in separate sections.

### 2.1 PTR Region

Gaseous H_2_O is introduced into a hollow cathode glow discharge reagent ion source. Due to the sophisticated design of the source, highly pure H_3_O^+^ is formed without the need for a mass filter and injected into the adjacent PTR reaction chamber (often depicted as “PTR drift tube”). Close to the original PTR‐MS setup, the reaction chamber consists of a series of metal ring electrodes with a resistor chain electrically connecting the individual rings. By applying a DC voltage across the resistor chain, a uniform electric field is created, which enables suppression of H_3_O^+^ clustering with H_2_O molecules in the sample air, while keeping analyte fragmentation low [1, 2]. Polytetrafluoroethylene (PTFE) spacers electrically isolate the individual ring electrodes and render the reaction region airtight. In common PTR reaction chambers, the pressure equilibrium between introducing sample air containing the analytes and pumping speed is set between 2 and 3 hPa. In the current prototype, this pressure is increased to 7.4 hPa to adjust to the subsequent SLIM IMS module. An ion funnel, as it is commonly used between the reaction region and the TOF analyzer [25], focuses the ions and thus considerably suppresses ion losses at the orifice to the SLIM IMS module.

### 2.2 SLIM IMS Region

The custom designed SLIM IMS module consists of two parallel printed circuit boards containing the electrodes for ion confinement (RF + DC), the traveling wave (TW) for ion transport (rectangular wave form) and the DC guard electrodes on each side. The board design comprises the following three main parts: (i) an ion accumulation area (7.5 cm)/a direct path, (ii) an injection port followed by a serpentine SLIM IMS drift path, and (iii) the loop path to allow ions to traverse several laps through the SLIM IMS drift path. The ion flow through each part is switched by multifunctional TW electrodes, working either as TW or as a gate. The gate voltage is set to approximately the same voltage as the guard voltage.

In TW based IMS, ions are separated by their arrival times at a detector. In order to measure these arrival times, one could simply inject a short “pulse” of ions into the IMS drift path and discard all ions until the next pulse. Obviously, this would result in an extremely low duty cycle and, thus, poor overall instrumental sensitivity. One countermeasure to considerably improve the duty cycle is to use sophisticated multiplexing methods for ion injection [24]. However, in our prototype, we decided on a different approach, namely, trapping the ions in an accumulation area (part i) prior to the injection point into the IMS drift path (part ii). This increases the load of ions injected at each injection in the form of an abundant ion package and allows accumulating the next package during the time of ion separation in the IMS drift path (e.g., 50 ms for 1 lap). In this mode, theoretically no ions are lost. While ion accumulation in TW based IMS is not new per se [26], it has, to the best of our knowledge, never been utilized in a PTR‐MS setup. The efficiency of this concept is discussed in the Results section.

Part i may be switched to direct path mode forming a short straight connection between the PTR reaction and the TOF region, that is, the instrument can be operated in “straight‐through” (continuous) mode without any IMS separation.

As mentioned before, if part (i) is operated in accumulation mode, the ions can be injected in packets into the SLIM IMS region (ii), which is made up of a series of parallel paths forming a serpentine path with a total length of 9 m. Via the switchable loop path (iii) the ions can be guided back into the serpentine path for an additional lap or forwarded to the TOF region. That is, the total path‐length can be freely extended by multiples of 9 m.

Most SLIM IMS devices reported in literature are designed for “heavy” ions with *m/z* in the region well above 300. Fundamental adaptations were necessary to ensure high sensitivity for “light” ions in the range between *m/z* 40 and 300, as typically found in PTR‐MS. One effective adaptation was “rounding” the turns of the serpentine drift path, that is, transforming the 180° square turns into semicircular turns, as suggested in the 2023 patent and 2024 publication of Deng et al. [27, 28]. Further improvements brought the selection of He instead of N_2_ as buffer gas and the increase of the RF confining frequency. This concept was recently validated by Frank et al. [29] with ions between *m/z* 270 and 514, and by Sabatini et al. [30] with ions down to *m/z* 182 (dinitrotoluenes).

For the evaluation measurements presented here, pure He as buffer gas, a TW frequency of 1.8 MHz with a TW amplitude ±22 V (1980 m/s “surf speed”), a pressure of 8 hPa, a SLIM RF confining frequency of 1.9 MHz, and a SLIM DC Guard voltage of 10 V were selected. To suppress ion losses at the transfer orifice, another ion funnel, similar to the one at the transition between the PTR reaction chamber and the SLIM IMS module, focuses the ions into the adjacent TOF analyzer.

### 2.3 TOF Region

The TOF analyzer is an ioniTOF 10k (IONICON Analytik GmbH., AT), which is of the orthogonal reflection type with a microchannel plate detector. The same type of analyzer was used and characterized by Reinecke et al. [7]. The maximum mass resolution achievable with this TOF device is about 15 000 m/∆m. However, as commonly known, maximum mass resolution comes with the tradeoff of reduced sensitivity, and vice versa. Thus, in the present study the resolution was tuned to about 12 000 m/∆m.

In the differential pumping region between the ion funnel of the SLIM IMS module and the pulsing region of the analyzer, an RF‐only hexapole ion guide minimizes ion losses due to dispersion and cools the translational motion of the ions for increased mass resolution.

### 2.4 Samples

The gas standard used for determining instrumental performance is a “Prüfgasgemisch” (test gas mixture; SIAD Austria GmbH., AT) containing about 1 ppmv (each) of various organic compounds in N_2_ matrix: acetone (C_3_H_6_O; protonated *m/z* 59.049), methyl vinyl ketone (C_4_H_6_O; protonated *m/z* 71.049); methyl ethyl ketone (C_4_H_8_O; protonated *m/z* 73.065); benzene (C_6_H_6_; protonated *m/z* 79.054); toluene (C_7_H_8_; protonated *m/z* 93.070), butyl methyl ketone (C_6_H_12_O; protonated *m/z* 101.096), p‐xylene (C_8_H_10_; protonated *m/z* 107.086), 2‐octanone (C_8_H_16_O; protonated *m/z* 129.127), and α‐pinene (C_10_H_16_; protonated *m/z* 137.133).

Furthermore, the headspaces of the following pure chemicals were used: C_5_H_10_O isomers (protonated *m/z* 87.080) 3‐methyl‐2‐butanone (Sigma‐Aldrich Chemie GmbH., Steinheim, DE; > 98.5%), 2‐pentanone (Sigma‐Aldrich Chemie GmbH., Steinheim, DE; > 99%). C_7_H_10_N_2_ isomers (protonated *m/z* 123.092) 2,3,5‐trimethylpyrazine (Sigma‐Aldrich Chemie GmbH., Steinheim, DE; 99%), 2‐ethyl‐3‐methylpyrazine (Sigma‐Aldrich Chemie GmbH., Steinheim, DE; > 98%), and 2‐ethyl‐5(6)‐methylpyrazine (Fluorochem Ltd., Hadfield, UK; 95%). In order to measure the samples, small flasks closed by caps with septa were prepared. The headspace samples were added to PTFE bags filled with zero air by piercing through the septa with an injection needle on a syringe.

For the proof‐of‐concept coffee headspace measurements, commercially available coffee beans were prepared using a bean‐to‐cup machine.

### 2.5 Data Processing

Integrated mass spectra (1‐s integration time) were processed with “PTR‐MS Viewer 3.4.7” (standard software for IONICON PTR‐TOF‐MS instruments). High time resolution SLIM IMS spectra were processed with proprietary software developed for the PTR‐SLIM‐TOF prototype.

IMS Resolution (Res) can be calculated based on the full width at half maximum (FWHM, “∆”) of a given peak in either an ion yield versus arrival time spectrum or an ion yield versus Collision Cross Section (CCS; compare [19]) spectrum. The CCS values depend on the buffer gas (He or N_2_) used. To allow comparison with other IMS devices, three IMS resolution metrics are reported here, ^time^Res_He_ (arrival time_He_/∆time_He_), ^CCS^Res_He_ (CCS_He_/∆CCS_He_), and ^CCS^Res_N2_ (CCS_N2_/∆CCS_N2_). ^time^Res_He_ is obtained directly from the arrival time spectra. As CCS values cannot be directly determined using TW based IMS (dynamic electric field), a calibration method is required. For this purpose, a calibration function of ^DT^CCS versus arrival time is created using drift tube ^DT^CCS values (static electric field). However, helium drift tube values ^DT^CCS_He_ for small molecules using similar ion sources are scarce. Thus, published reduced helium ion mobility values ^DT^K_0_He_ [31] of 2‐pentenal, pentanal, 2‐heptenal, heptanal, 2‐octenal, and octanal were used. The corresponding collision cross sections ^DT^CCS_He_ are calculated using the fundamental low‐field ion mobility equation as recommended by Gabelica et al. [32] (Table S1, Formula S1). These samples were measured to create a calibration function to determine ^SLIM^CCS_He_ values of benzene 47.7 ± 0.9 ${Å}^{2}$, toluene 53.1 ± 1.0 ${Å}^{2}$, and p‐xylene 58.9 ± 1.0 ${Å}^{2}$. These three compounds were chosen because of their ready availability in gas standards. Linear calibration functions were then created for each measurement using the ^SLIM^CCS_He_ values of benzene, toluene, and p‐xylene. While power law or polynomial functions are commonly used for this purpose, the data is well described linearly for this narrow CCS range (Figure S2). Using this calibration procedure, ^CCS^Res_He_ values were calculated for each compound. While this approach for deriving absolute CCS values of uncalibrated compounds may introduce some error, the relative value ^CCS^Res_He_ (CCS_He_/∆CCS_He_) remains reasonably accurate for meaningful comparison with other IMS devices. Particularly, as strong correlation between calibrated (TW IMS) and measured (drift tube IMS) CCS values has already been demonstrated [29].

As most IMS measurements are conducted using $N_{2}$ buffer gas, we also provide the corresponding nitrogen IMS resolution values ^CCS^Res_N2_ derived from the helium‐based measurements. These are serving only the purpose of enabling resolution comparison with other IMS devices. Thus, for each measurement an additional linear calibration function is created using the CCS_N2_ values of benzene 111.7 ± 1.1 ${Å}^{2}$, toluene 115.3 ± 1.2 ${Å}^{2}$, and p‐xylene 119.2 ± 1.2 ${Å}^{2}$, directly available from the CCSbase website (Figure S2) [33, 34].

## 3 Results

**FIGURE 2:**
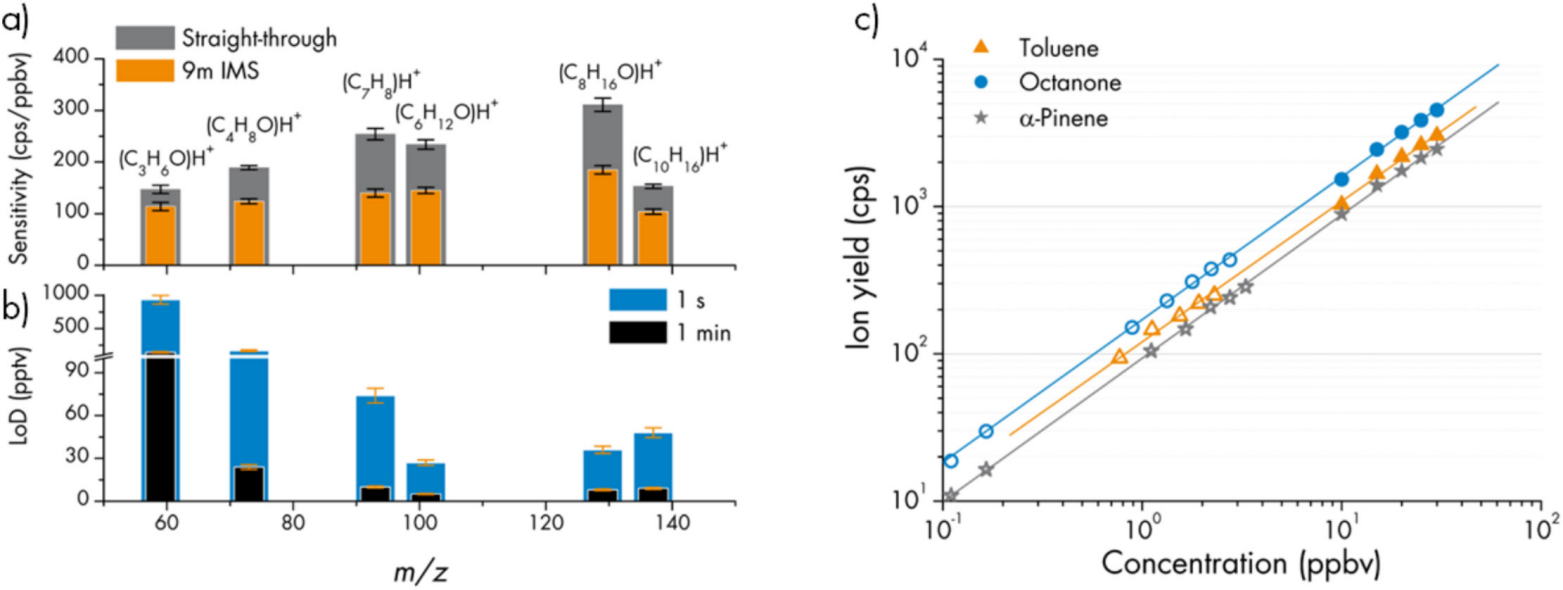
(a) Sensitivity comparison between (continuous) straight‐through mode and ion accumulation followed by one SLIM IMS lap for six exemplary compounds in the gas standard (acetone (C_3_H_6_O), methyl ethyl ketone (C_4_H_8_O), toluene (C_7_H_8_), butyl methyl ketone (C_6_H_12_O), 2‐octanone (C_8_H_16_O), and α‐pinene (C_10_H_16_)). (b) LoDs in SLIM IMS mode for these six compounds, after 1 s and 1 min data integration time. (c) Linearity of ion yield vs. analyte concentration in SLIM IMS mode for toluene, octanone, and α‐pinene.

First, we compare the measured sensitivities of selected compounds in the gas standard between the (continuous) “straight‐through” and the (pulsed) SLIM IMS mode. In doing so, the efficiency of the ion accumulation, as well as “losslessness” (SLIM) of ion migration along the 9‐m serpentine drift path can be quantified. In Figure 2a, the results are shown, which range between 78% (22% losses; protonated acetone at nominal *m/z* 59) and 55% (45% losses; protonated toluene at nominal *m/z* 93) accumulation/IMS efficiency. On average, about 65% of the ions that reach the TOF detector in straight‐through mode are still detected after being accumulated and sent through the 9‐m IMS serpentine. This is a higher percentage than what was previously reported for a 9‐m IMS serpentine with multiplexed ion injection (40% [24]), confirming the new milestone in using SLIM IMS and allowing us to potentially extend the IMS path to higher values. It should be noted that for the data displayed in Figure 2a, only for α‐pinene a noteworthy fragmentation (about 30% to nominal *m/z* 81) was observed. This explains the low absolute values of protonated α‐pinene (nominal *m/z* 137) sensitivity, but has no effect on the stated efficiency, which is a relative value. Furthermore, we did not observe any notable differences in product ion ratios (*m/z* 137: *m/z* 81) between straight‐through and IMS mode. In both modes, the ratio was 70:30, which corresponds to a reduced electric field (E/N) in the PTR reaction chamber of about 70 Td according to literature values [35]. Calculating the E/N with a reaction chamber pressure of 7.4 hPa and 800 V DC applied to the chamber electrodes confirms the value of about 70 Td. Thus, we conclude that no or negligible additional ion fragmentation occurs in the SLIM IMS module.

Using these sensitivity values, the limits‐of‐detection (LoDs) can be calculated via 3 times the standard deviation of the signal when zero air is sampled (chemical background). In Figure 2b, the results are shown for 1 s and for 1‐min signal integration time in SLIM IMS mode. Unfortunately, our zero air contained about 5 ppbv acetone, which explains the unusually high LoD of this compound. By using the prototype configuration, the 1 s and 1‐min SLIM IMS LoDs for protonated butyl methyl ketone, octanone and α‐pinene are well below 50 pptv and 10 pptv, respectively. It should be noted that with recent developments in enhancing the sensitivity of PTR‐MS and coupling this with SLIM IMS, these values can be easily improved in commercial instruments.

Finally, the exemplary data of toluene, octanone, and α‐pinene in Figure 2c indicate that the signal response for the accumulation/SLIM IMS mode is linear over several orders of magnitude, as it is expected from common PTR‐MS. The data points were obtained by analyzing the gas standard at five dilution steps and including the isotope signals at nominal *m/z* 94, 130, and 138, for toluene, octanone, and α‐pinene, respectively. For octanone and α‐pinene, the isotope signals at nominal m/z 131 and 139 of the two highest concentrations are also included.

**FIGURE 3:**
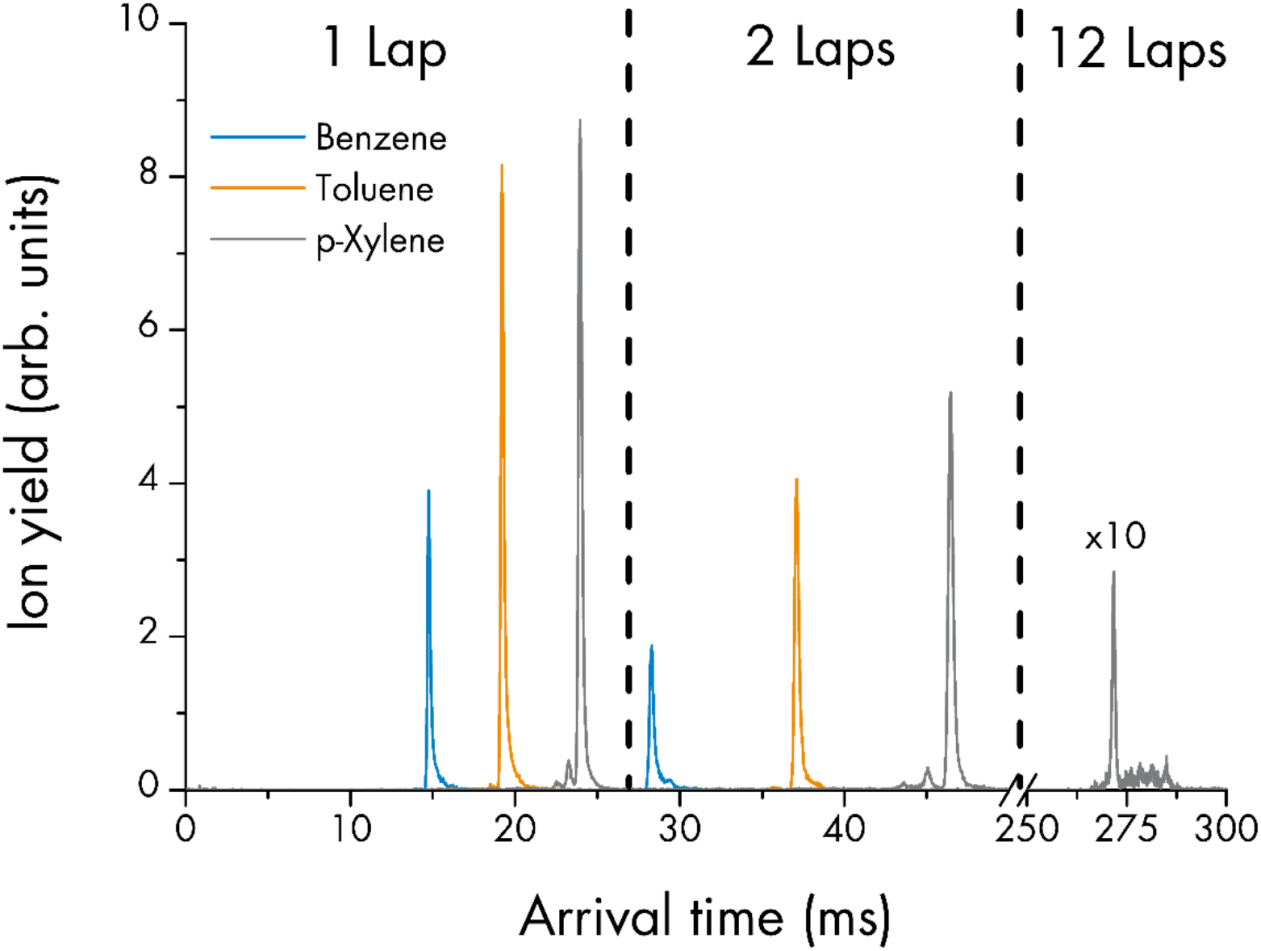
IMS spectra for benzene, toluene, and p‐xylene for one and two SLIM IMS laps, respectively. Additionally, for p‐xylene the spectrum for 12 SLIM IMS laps is shown (signal multiplied by a factor of 10).

So far, we have confirmed that the main PTR‐MS benefits, sensitivity, linearity, and LoDs, are largely preserved in SLIM IMS mode. The main advantage of this mode is the addition of another data dimension to the TOF mass spectra. For a series of *m/z* corresponding to compounds from the gas standard, exemplary IMS spectra are shown in Figure S1. However, for a more detailed discussion we selected the compounds used for CCS calibration functions. Figure 3 shows the separation of benzene (^SLIM^CCS_He_ 47.7 ± 0.9 ${Å}^{2}$), toluene (^SLIM^CCS_He_ 53.1 ± 1.0 ${Å}^{2}$), and p‐xylene (^SLIM^CCS_He_ 58.9 ± 1.0 ${Å}^{2}$) after one and two laps. Additionally, a measurement of p‐xylene after 12 laps is included. This figure visualizes the high IMS resolving power increasing with multiple laps. The ∆time_He_, ^time^Res_He_, ^CCS^Res_He_, and ^CCS^Res_N2_ of all peaks depicted are presented in Table 1. The arrival time difference between benzene and toluene increases from 4.45 ms in the first lap to 8.80 ms in the second lap and the arrival time difference between toluene and p‐xylene from 4.75 ms in the first lap to 9.35 ms in the second lap. High IMS resolution is achieved if the arrival time difference of one component to the others increases while the ∆time_He_ stays narrow. This is quantified by a single value, ^CCS^Res. For p‐xylene, ^CCS^Res_He_ is already 190 in the first lap and rises to 276 in the second lap. By the 12th lap, the ^CCS^Res_He_ reaches 540. As the IMS resolution in the first lap may be sufficient for most applications, the measurement after the 12th lap, corresponding to a path length of more than 100 m, mainly demonstrates the low ion loss over extended travel.

**TABLE 1:** IMS resolution data for different numbers of SLIM IMS laps.

Compound	Chemical formula	Lap	∆Time_He_ (ms)	^time^Res_He_	^CCS^Res_He_	^CCS^Res_N2_
Benzene	(C_6_H_6_)H^+^	1	0.21	71	188	660
Toluene	(C_7_H_8_)H^+^	1	0.23	84	189	610
p‐Xylene	(C_8_H_1__0_)H^+^	1	0.26	94	190	570
Benzene	(C_6_H_6_)H^+^	2	0.32	89	245	860
Toluene	(C_7_H_8_)H^+^	2	0.31	118	274	890
p‐Xylene	(C_8_H_1__0_)H^+^	2	0.35	134	276	830
p‐Xylene	(C_8_H_1__0_)H^+^	12	0.94	288	540	1630

Furthermore, the lap time, the time a component takes for each subsequent lap after the first, is for benzene 13.52 ms, for toluene 17.87 ms, and for p‐xylene 22.49 ms.

**FIGURE 4:**
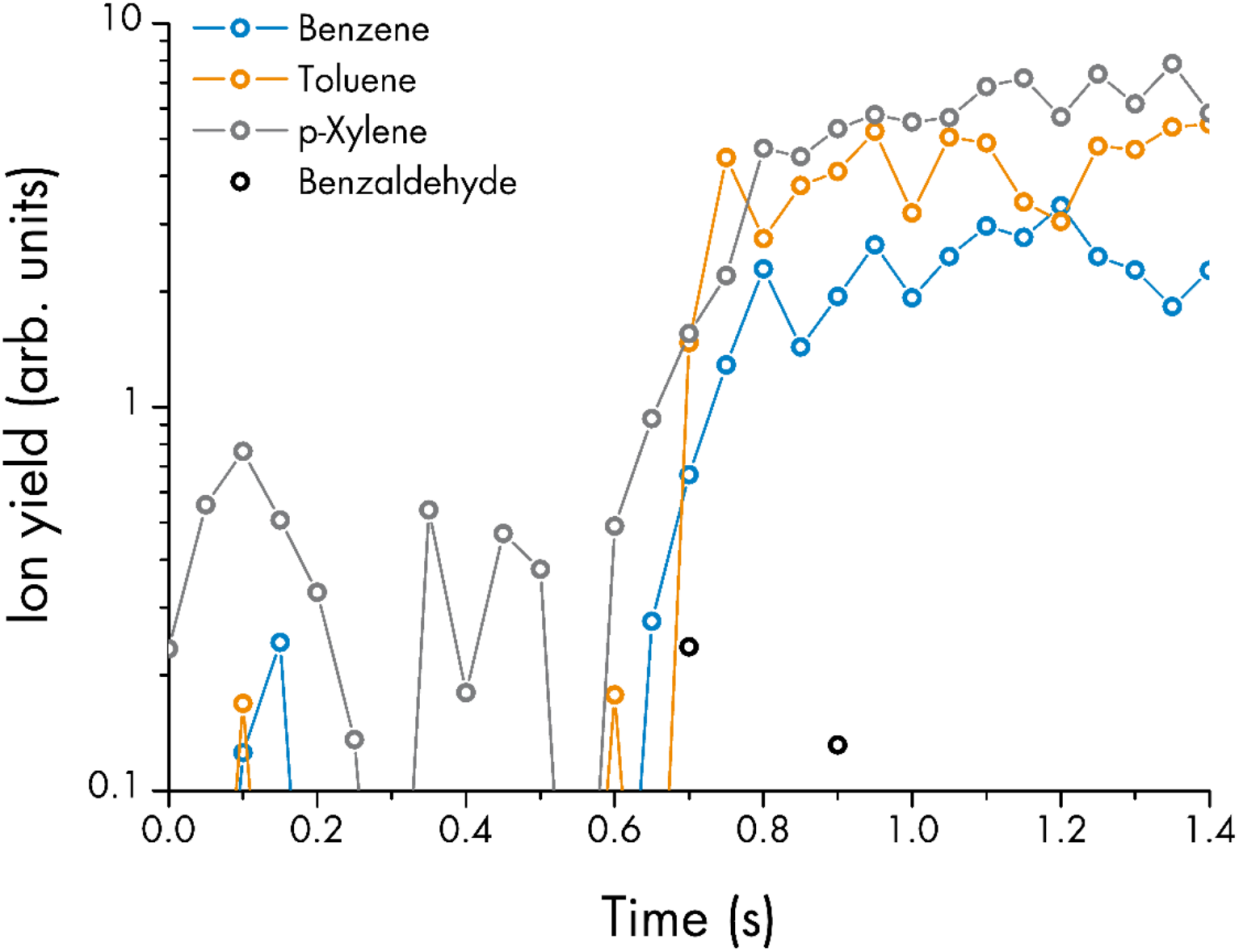
Signal response when connecting the gas standard, recorded at a time resolution of 50 ms. *Note:* Benzaldehyde was not present in the gas standard and is shown to demonstrate that no signal crosstalk occurs.

The last of the crucial performance parameters to be evaluated for the PTR‐SLIM‐TOF prototype is the response time. This will answer the question if the instrument's real‐time capabilities are preserved. The SLIM IMS was set to 1 lap per ion package. For each of the compounds benzene, toluene, and p‐xylene, the respective IMS peaks were quantified using the IMS arrival times obtained in the previous experiment. The minimum time resolution between data points is 50 ms, which is the time for one SLIM IMS cycle to be completed (compare Figure S1). Figure 4 shows a close‐up of these data points (interconnected with straight lines for better visibility) versus time around the moment when the instrument's sampling line was switched from zero air to the gas standard. This switching was performed by presetting the gas standard flow to prevent overshooting of the mass flow controller upon activation and quickly connecting the standard to the zero airline via T‐piece. It can be seen that within 3–5 data points about 90% of the final signal intensity is reached for all three compounds. The response times therefore are between 150 and 250 ms. While the exact values are of lesser importance, these data support the conclusion that PTR‐SLIM‐TOF provides a new level of PTR‐MS selectivity indeed in real‐time.

In Figure 4 in addition to p‐xylene (protonated *m/z* 107.086), also data points corresponding to benzaldehyde (protonated *m/z* 107.049; not present in the gas standard) are displayed. With an *m/z* difference of only 0.037, these two isobars can show some crosstalk when quantified from mass spectral data. In cases where the signal intensity of one isobar is orders of magnitude higher than the other, this can even be the case for high‐end mass resolution TOF analyzers. It can be seen that there is definitely no crosstalk between p‐xylene and benzaldehyde with additional IMS separation, as the latter only shows two data points above the threshold, which most probably originate from signal noise.

**FIGURE 5:**
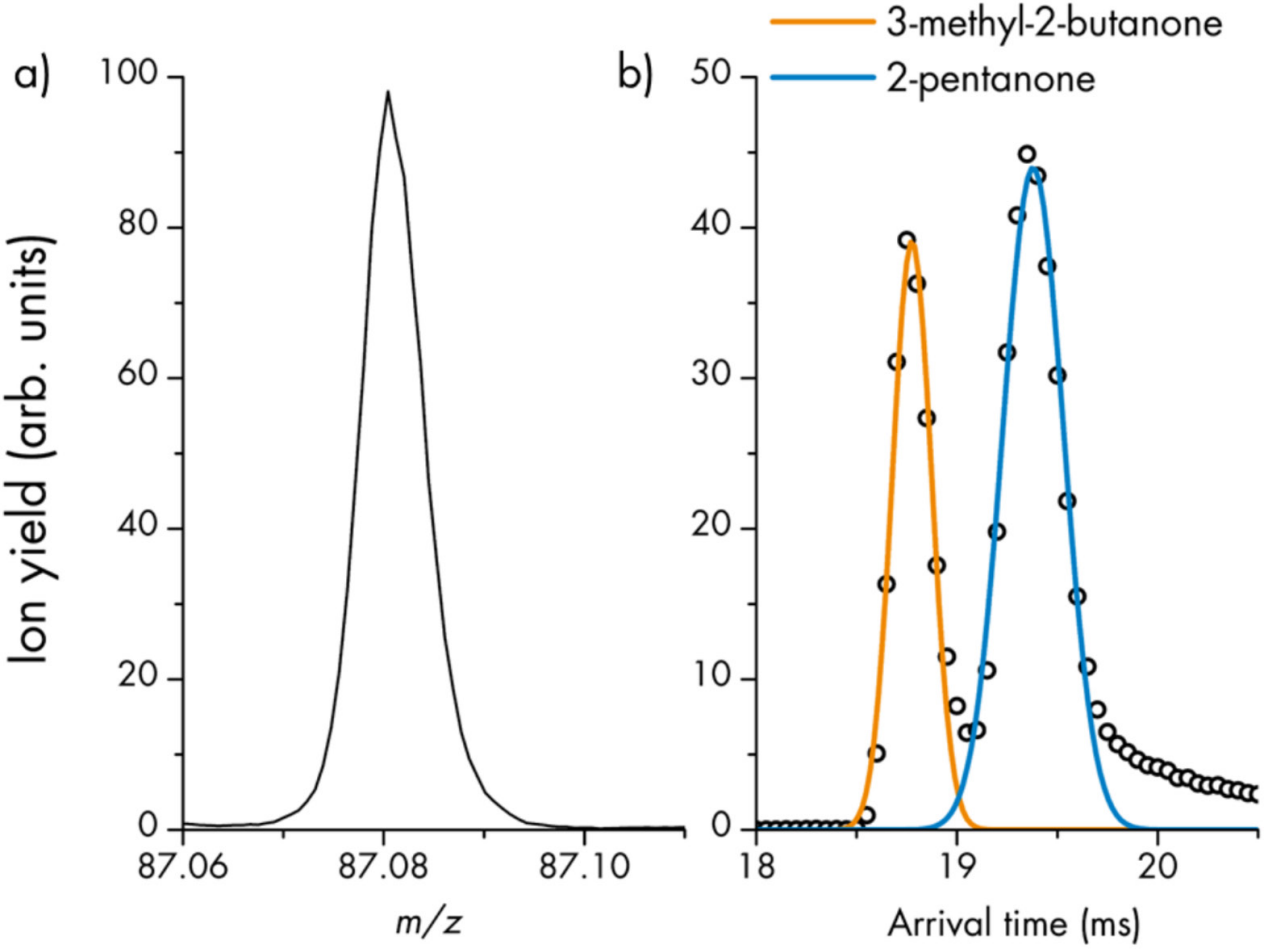
(a) Section of a mass spectrum around m/z 87.080 ((C_5_H_10_O)H^+^). (b) IMS spectrum (1 lap) between 18 and 21 ms arrival time for m/z 87.080 with two Gaussian fits corresponding to the respective isomers.

The ultimate goal of PTR‐MS selectivity, however, is the separation of isomeric compounds. The isomers 3‐methyl‐2‐butanone and 2‐pentanone share the sum formula C_5_H_10_O. Therefore, the protonated molecules of both compounds appear at *m/z* 87.080 in a PTR‐TOF‐MS mass spectrum and are indistinguishable. Figure 5a shows this single mass spectral peak with a FWHM resolution of about 12 000 m/∆m, that is, the resolution that is expected from this type of TOF analyzer. For many applications this result can be unsatisfying, as in stark contrast to the solvent 3‐methyl‐2‐butanone, 2‐pentanone is also a prominent flavor compound [36].

The problem is solved in Figure 5b, which displays the IMS data, including Gaussian fits for the two observed peaks. The two isomers were identified by individually manipulating the introduced concentrations and are clearly separated close to the baseline, with a valley of about 15% of the average signal height. The ^time^Res_He_ of the 3‐methyl‐2‐butanone and 2‐pentanone peaks is 78 and 53, respectively. Applying the calibration method described in the Experimental section, this corresponds to ^CCS^Res_He_ 121–179 and ^CCS^Res_N2_ 390–590. In Table 2, the various Res values are shown, including those from experiments utilizing multiple SLIM IMS laps. The second lap increases the resolution by 40%–45%. Adding a third lap results in a further increase of 12%–17%.

**TABLE 2:** Different Res values for the two isomers 3‐methyl‐2‐butanone and 2‐pentanone for one to three SLIM IMS laps, respectively.

Compound	Chemical formula	Lap	^time^Res_He_	^CCS^Res_He_	^CCS^Res_N2_
3‐Methyl‐2‐butanone	(C_5_H_10_O)H^+^	1	78	179	590
2‐Pentanone	(C_5_H_10_O)H^+^	1	53	121	390
3‐Methyl‐2‐butanone	(C_5_H_10_O)H^+^	2	110	258	850
2‐Pentanone	(C_5_H_10_O)H^+^	2	77	177	570
3‐Methyl‐2‐butanone	(C_5_H_10_O)H^+^	3	123	294	960
2‐Pentanone	(C_5_H_10_O)H^+^	3	90	210	680

Because the analysis of headspace above freshly brewed coffee was one of the first PTR‐MS applications, already mentioned in the 1998 publication by Lindinger et al. [2], we chose this sample for a first proof‐of‐concept real‐life test of the PTR‐SLIM‐TOF prototype setup. When researchers switched from the nominal mass resolution of quadrupole mass filters to the isobar separating resolution of TOF analyzers, they used the term “rich information” to describe the data acquired from food samples [37]. Now, with one full IMS spectrum for each of the (mostly) multiple peaks per nominal *m/z* in a high‐resolution TOF mass spectrum of coffee headspace (compare Figure S3 + Figure S4), this information might be described as “opulent” (Figure S5). Virtually every isobar peak, which until now has all too often been attributed to a single substance in PTR‐MS, is in reality composed of a whole series of isomeric compounds. This fact has been well known to the GC–MS community. However, with PTR‐SLIM‐TOF these data are now for the first time available in real‐time, with a response time in the 10^2^‐millisecond region. It should be noted that even very small molecules, like, for example, C_2_H_4_O (protonated *m/z* 45.034; most probably acetaldehyde), can be detected in the coffee spectra (Figure S3a + Figure S5). This is remarkable for the SLIM IMS technology, which was originally demonstrated for large molecules, as previously mentioned.

**FIGURE 6:**
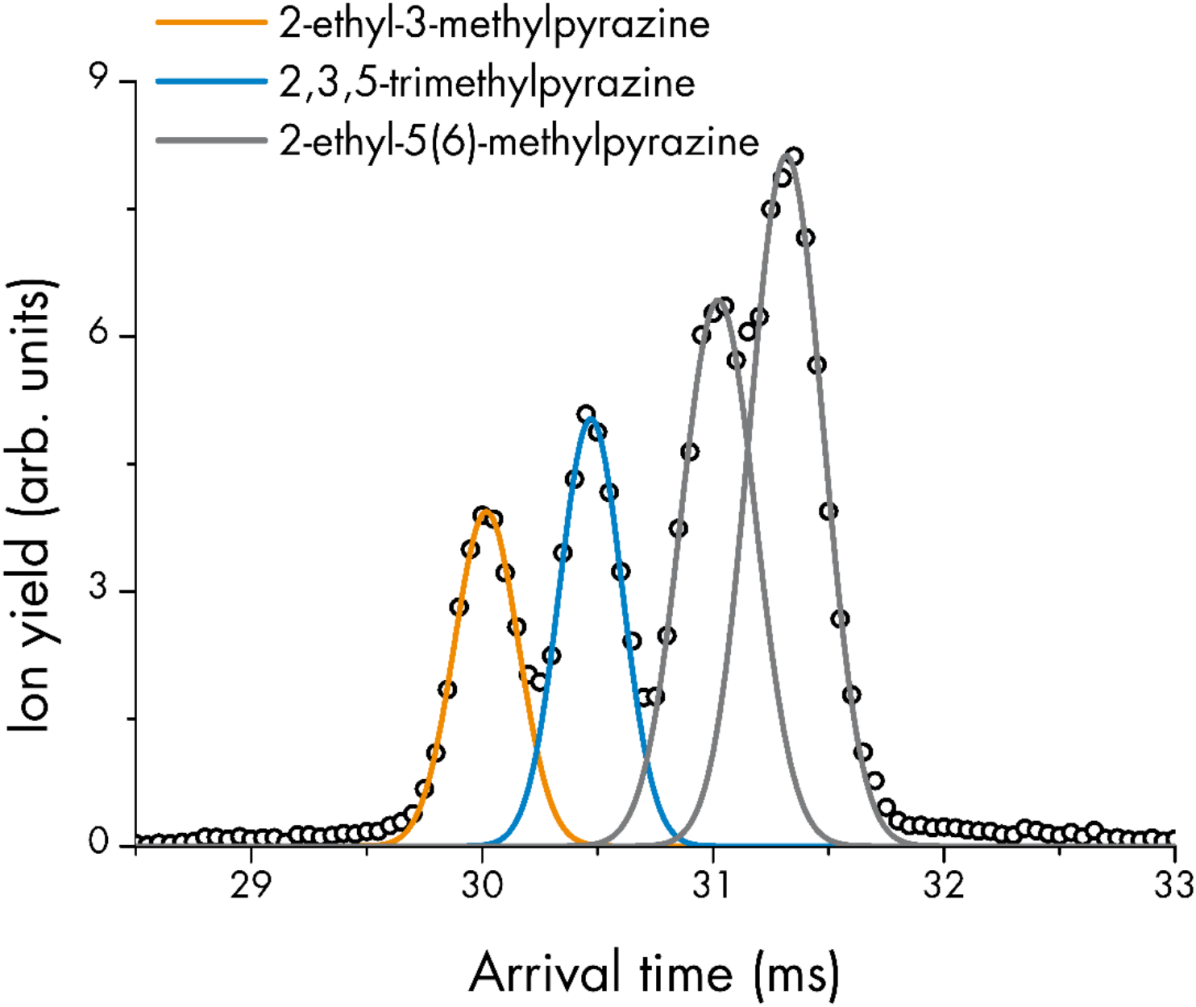
IMS spectrum for m/z 123.092 with Gaussian curves fitted to the data. *Note:* 2‐Ethyl‐5‐methylpyrazine and 2‐ethyl‐6‐methylpyrazine could not be assigned to the individual peaks, as both isomers were in the standard used for identification. Trajectory calculations could help to resolve this issue.

Analyzing and identifying all signals in the PTR‐SLIM‐TOF spectra would be well beyond the scope of this proof‐of‐concept study. Therefore, we only discuss the IMS spectrum for *m/z* 123.092 in Figure 6 (for the corresponding section of the mass spectrum see Figure S4b) as an example. Fitting Gaussian curves to the data reveals four peaks, with the first two being very well separated (valley < 50% max. signal height) and the last two somewhat overlapping, but still separable. By successively adding the individual pure substances to the coffee headspace as internal standards, the first two peaks could be identified as 2‐ethyl‐3‐methylpyrazine and 2,3,5‐trimethylpyrazine, respectively. Unfortunately, for 2‐ethyl‐5‐methylpyrazine and 2‐ethyl‐6‐methylpyrazine, only an isomer mixture standard was available (2‐ethyl‐5(6)‐methylpyrazine), which reproduced the partly overlapping third and fourth peak structure. Therefore, no assignment of the two peaks to the individual isomers is possible at this time. However, all four pyrazines are prominent examples for coffee flavor compounds [38] that so far could only be quantified in real‐time as a sum of all isomers with PTR‐TOF‐MS or as individual isomers with offline GC (pre)separation. The example indicates that PTR‐SLIM‐TOF has the potential to combine the benefits of both technologies and provide isomer specific quantification in real‐time. In cases where a separated standard is not available, ion mobility collision cross sections can be calculated with the trajectory method, using codes like IMoS [39] or MassCCS [40, 41 ]. To assign also 2‐ethyl‐5‐methylpyrazine and 2‐ethyl‐6‐methylpyrazine, one would calculate collision cross sections for all four *m/z* 123.092 isomers. If 2‐ethyl‐3‐methylpyrazine and 2,3,5‐trimethylpyrazine are correctly calculated as the isomers with the smallest CCS values, it can be assumed that the relative CCS values of the unassigned isomers are correctly predicted. It should be noted that this approach may not always produce correct results.

## 4 Summary and Conclusion

Combining PTR‐TOF‐MS with SLIM IMS represents a significant enhancement in selectivity, enabling the differentiation of isomeric compounds and improving the separation capability of isobaric compounds while preserving the real‐time capabilities of PTR‐MS. The ability of SLIM IMS to separate isomers in a timescale of milliseconds offers an advantage over gas chromatography (GC), the current gold standard in separating isomers in minutes. This has far‐reaching potential for many PTR‐MS applications, including atmospheric science, food science, medical applications, and the semiconductor industry.

We showed that with several adaptations we successfully found a solution to use the SLIM design for “light” ions in the range below *m/z* 300, that are typically analyzed using PTR‐MS. A helium‐based ion mobility resolution CCS_He_/∆CCS_He_ of about 200 could be observed in a single lap, with a maximum of about 500 for more than 100 m IMS drift path, corresponding to a nitrogen‐based ion mobility resolution CCS_N2_/∆CCS_N2_ of 600 and 1600, respectively.

To demonstrate the applicability of the high ion mobility resolution of the SLIM device, the protonated isomeric system of 3‐methyl‐2‐butanone and 2‐pentanone at *m/z* 87.080 was separated in up to three laps, highlighting the improved separation achieved through multiple laps and confirming the outstandingly high resolution already achieved for one lap. The limit of detection LOD of the prototype was quantified and corresponds to expected (from state‐of‐the‐art PTR‐MS) values ranging a few tenths of pptv. The obtained sensitivity values and the fact that after 12 IMS laps still some signal could be detected emphasize the minimal loss of ions (less than 40%) when using the IMS separation in contrast to direct transfer (straight‐through). First measurements by using a “real‐life” sample like freshly brewed coffee show the full potential of the new method. Key coffee flavor compounds 2‐ethyl‐3‐methylpyrazine, 2,3,5‐trimethylpyrazine, and 2‐ethyl‐5(6)‐methylpyrazine, which are isomers of C_7_H_10_N_2_, could be clearly separated.

In summary, we believe that PTR‐SLIM‐TOF has been shown to solve most of the PTR‐MS selectivity problems mentioned in the Introduction without introducing any disadvantages. In particular, the real‐time capability and most of the sensitivity of the technology are retained.

## Conflicts of Interest

IONICON Analytik is engaged in the commercialization of PTR‐SLIM‐TOF technology.

## Supporting information


**Table S1:** Drift tube K_0_ and CCS values.
**Figure S1:** Six IMS spectra corresponding to compounds in the gas standard. Different colors correspond to ion yields detected for different mass spectral peaks (labels = *m/z* of the peaks' centers).
**Figure S2:** CCS calibration curves for He and N_2_, for 1, 2, and 12 laps, respectively.
**Figure S3:** Averaged mass spectrum of coffee headspace (averaged over 200 s): (a) *m/z* 18–100.5, (b) *m/z* 100.5–200.5. Peaks used for IMS spectra in Figure S5 are labelled.
**Figure S4:** Mass spectra sections for: (a) isobaric compounds at nominal *m/z* 107, (b) system from Figure 6 at nominal *m/z* 123, and (c) isobaric compounds at nominal *m/z* 153. Fitted peaks are labeled with their exact *m/z* and a tentative identification.
**Figure S5:** Coffee headspace IMS spectra for selected *m/z*. Upper graph = linear, lower graph = log y‐axis. **Formula S1:** Calculation ofCCSfromK_0_.

## Data Availability

The PTR‐SLIM‐TOF prototype generates a proprietary data format. Extracted ASCII data are available by request from the corresponding author.
