# Supplementary material for: Selectivity Beyond Mass: Real‐Time Isomer Separation Using SLIM IMS Coupled to PTR‐MS
Source: J Mass Spectrom. 2026 Jan 28;61(2):e70031. doi: 10.1002/jms.70031 (PMC12853077; doi:10.1002/jms.70031)
Supplement: Supplementary file 1 — Table S1: Drift tube K0 and CCS values. Figure S1: Six IMS spectra corresponding to compounds in the gas standard. Different colors correspond to ion yields detected for different mass spectral peaks (labels = m/z of the peaks' centers). Figure S2: CCS calibration curves for He and N2, for 1, 2, and 12 laps, respectively. Figure S3: Averaged mass spectrum of coffee headspace (averaged over 200 s): (a) m/z 18–100.5, (b) m/z 100.5–200.5. Peaks used for IMS spectra in Figure S5 are labelled. Figure S4: Mass spectra sections for: (a) isobaric compounds at nominal m/z 107, (b) system from Figure 6 at nominal m/z 123, and (c) isobaric compounds at nominal m/z 153. Fitted peaks are labeled with their exact m/z and a tentative identification. Figure S5: Coffee headspace IMS spectra for selected m/z. Upper graph = linear, lower graph = log y‐axis. Formula S1: Calculation ofCCSfromK 0. [file JMS-61-e70031-s001.docx]

**Supporting Information**

Selectivity Beyond Mass: Real-Time Isomer Separation using SLIM IMS coupled to PTR-MS

Jacob Jordan^1,2^, Alfons Jordan^1^, Christian Lindinger^1^, Gernot Hanel^1^, Tobias Fügenschuh^1^, Martin K. Beyer^2,*^, Philipp Sulzer^1,**^

^1^ IONICON Analytik GmbH., Eduard-Bodem-Gasse 3, 6020 Innsbruck, AUSTRIA

^2^ Institut für Ionenphysik und Angewandte Physik, Universität Innsbruck, Technikerstraße 25/3, 6020 Innsbruck, AUSTRIA

^*^ [Martin.Beyer@uibk.ac.at](mailto:Martin.Beyer@uibk.ac.at)

^**^ [philipp.sulzer@ionicon.com](mailto:philipp.sulzer@ionicon.com)

**Content**

Formula S1: Calculation of CCS from K_0._

Table S1: Drift tube K_0_ and CCS Values.

Figure S1: Six IMS spectra corresponding to compounds in the gas standard. Different colors correspond to ion yields detected for different mass spectral peaks (labels = *m/z* of the peaks' centers).

Figure S2: CCS calibration curves for He and N_2_, for 1, 2 and 12 laps, respectively.

Figure S3: Averaged mass spectrum of coffee headspace (averaged over 200 s): a) *m/z* 18 – 100.5, b) *m/z* 100.5 – 200.5. Peaks used for IMS spectra in Figure S5 are labelled.

Figure S4: Mass spectra sections for: a) isobaric compounds at nominal *m/z* 107, b) system from Fig. 6 at nominal *m/z* 123, c) isobaric compounds at nominal *m/z* 153. Fitted peaks are labeled with their exact *m/z* and a tentative identification.

Figure S5: Coffee headspace IMS spectra for selected *m/z*. Upper graph = linear, lower graph = log y-axis.

References

Formula S1: Calculation of CCS from K_0._

$$CCS=\frac{3}{16}\sqrt{\frac{2\pi}{\mu k_{B}T}} \frac{ze}{N_{0}K_{0}}$$

where $N_{0}$ is the Loschmidt’s number, $k_{B}$ is the Boltzmann constant, *z* is the absolute charge of the ion, *T* is the temperature, *e* is the elementary charge, $K_{0}$ is the reduced ion mobility and *μ* is the reduced mass of the ion-buffer gas pair

$$\mu=\frac{m_{i}m_{g}}{m_{i}+m_{g}}$$

where $m_{i}$ is the mass of the ion and $m_{g}$ the mass of the buffer gas.

Table S1: Drift tube K_0_ and CCS Values.

| **Compound** | **protonated m/z** | **Chemical**  **Formula** | **^DT^K₀_, He_ (cm² V⁻¹ s⁻¹) [^[[1]](#endnote-1)^]** | **^DT^CCS_He_ (Å²)** |
| --- | --- | --- | --- | --- |
| water | 19 | H₃O⁺ | 21.80 ± 0.09 | 27.14 ± 0.11 |
| 2-pentenal | 85 | (C₅H₈O)H⁺ | 10.60 ± 0.17 | 51.91 ± 0.83 |
| pentanal | 87 | (C₅H₁₀)H⁺ | 10.30 ± 0.07 | 53.40 ± 0.37 |
| 2-heptenal | 113 | (C₇H₁₂O)H⁺ | 8.40 ± 0.08 | 65.14 ± 0.65 |
| heptanal | 115 | (C₇H₁₄O)H⁺ | 8.70 ± 0.09 | 62.88 ± 0.63 |
| 2-octenal | 127 | (C₈H₁₄O)H⁺ | 7.50 ± 0.17 | 72.8 ± 1.6 |
| octanal | 129 | (C₈H₁₆O)H⁺ | 7.90 ± 0.03 | 69.12 ± 0.28 |

Figure S1: Six IMS spectra corresponding to compounds in the gas standard. Different colors correspond to ion yields detected for different mass spectral peaks (labels = *m/z* of the peaks' centers).


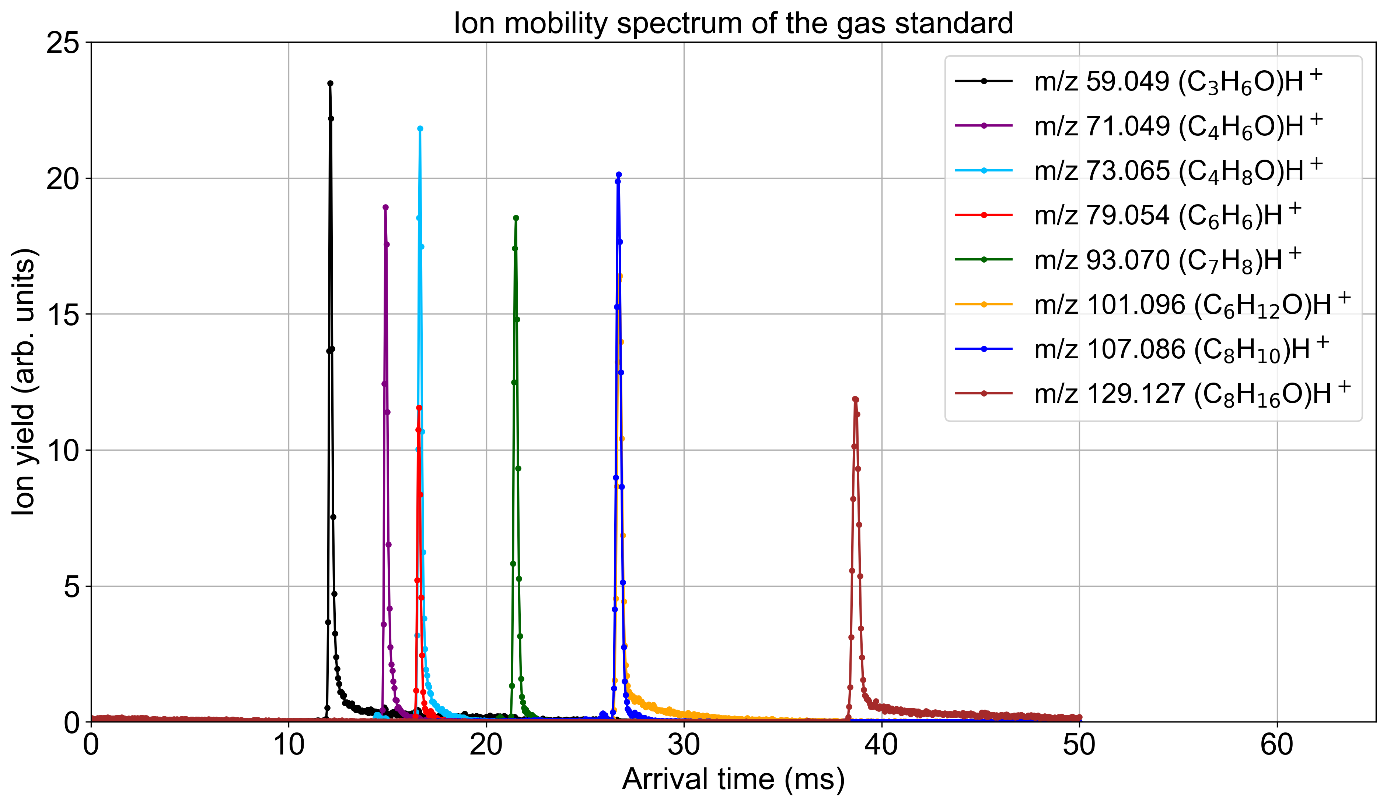


Figure S2: CCS calibration curves for He and N_2_, for 1, 2 and 12 laps, respectively.

| 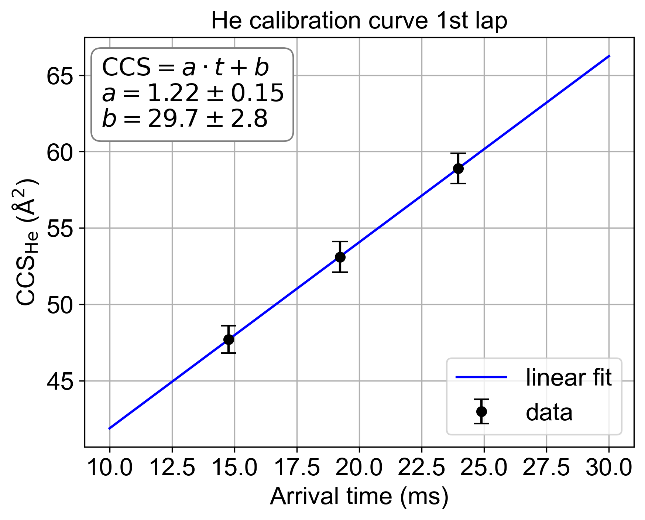 | 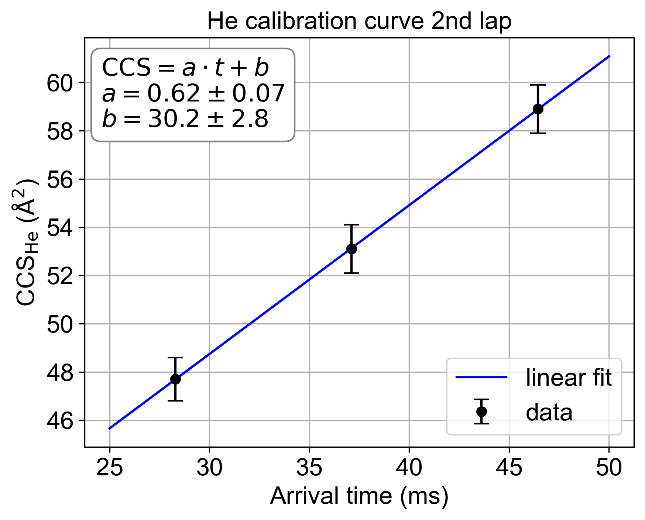 |
| --- | --- |
| 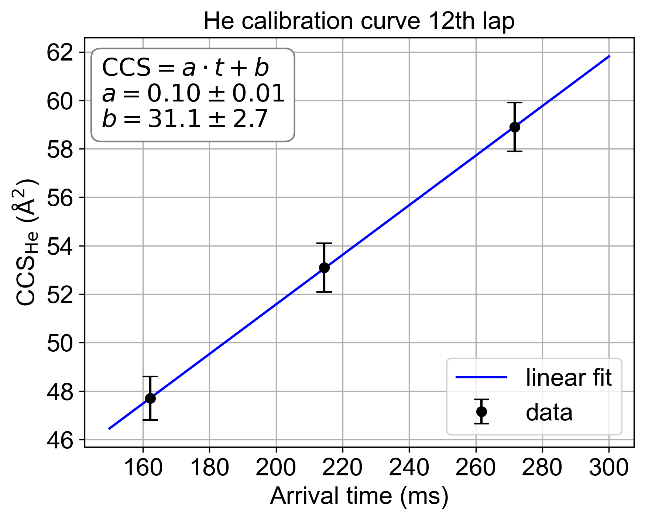 | |
| 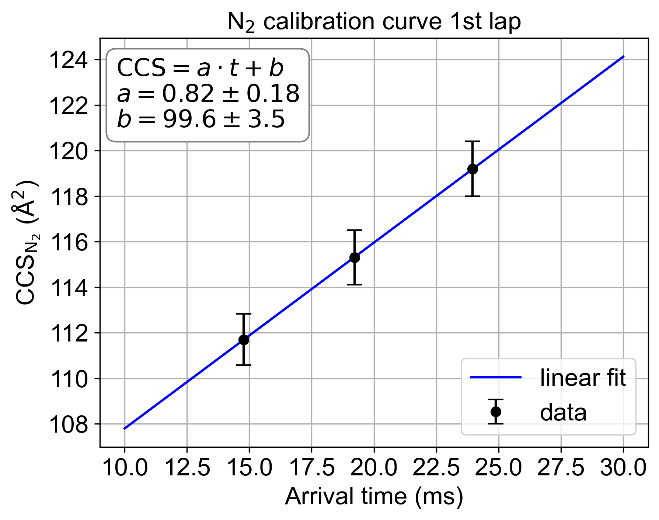 | 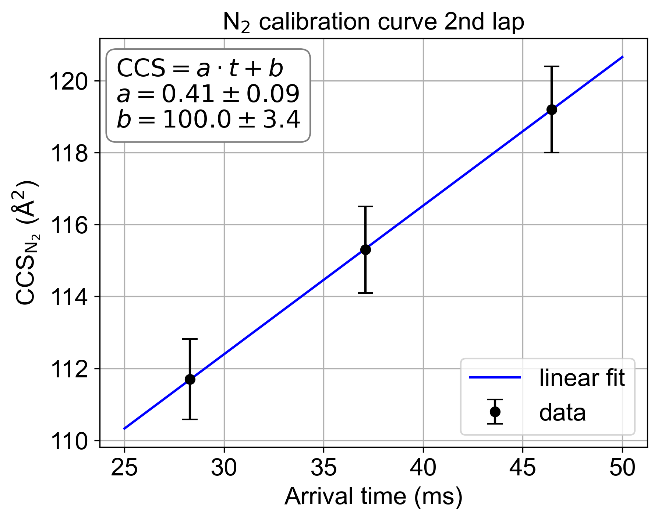 |
| 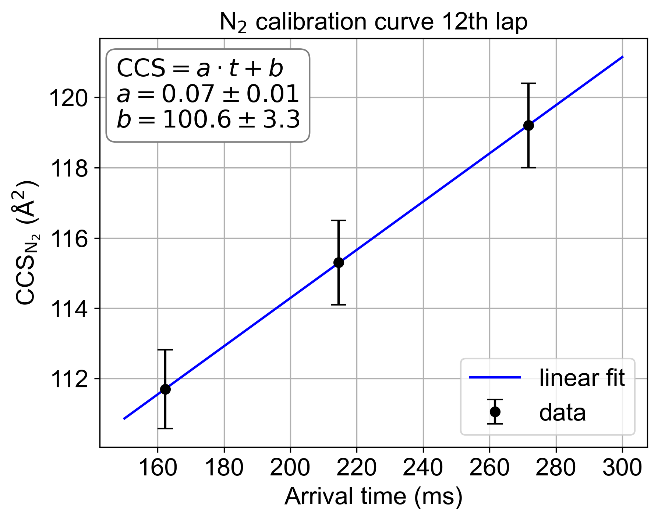 | |

Figure S3: Averaged mass spectrum of coffee headspace (averaged over 200 s): a) *m/z* 18 – 100.5, b) *m/z* 100.5 – 200.5. Peaks used for IMS spectra in Figure S5 are labelled.

a)


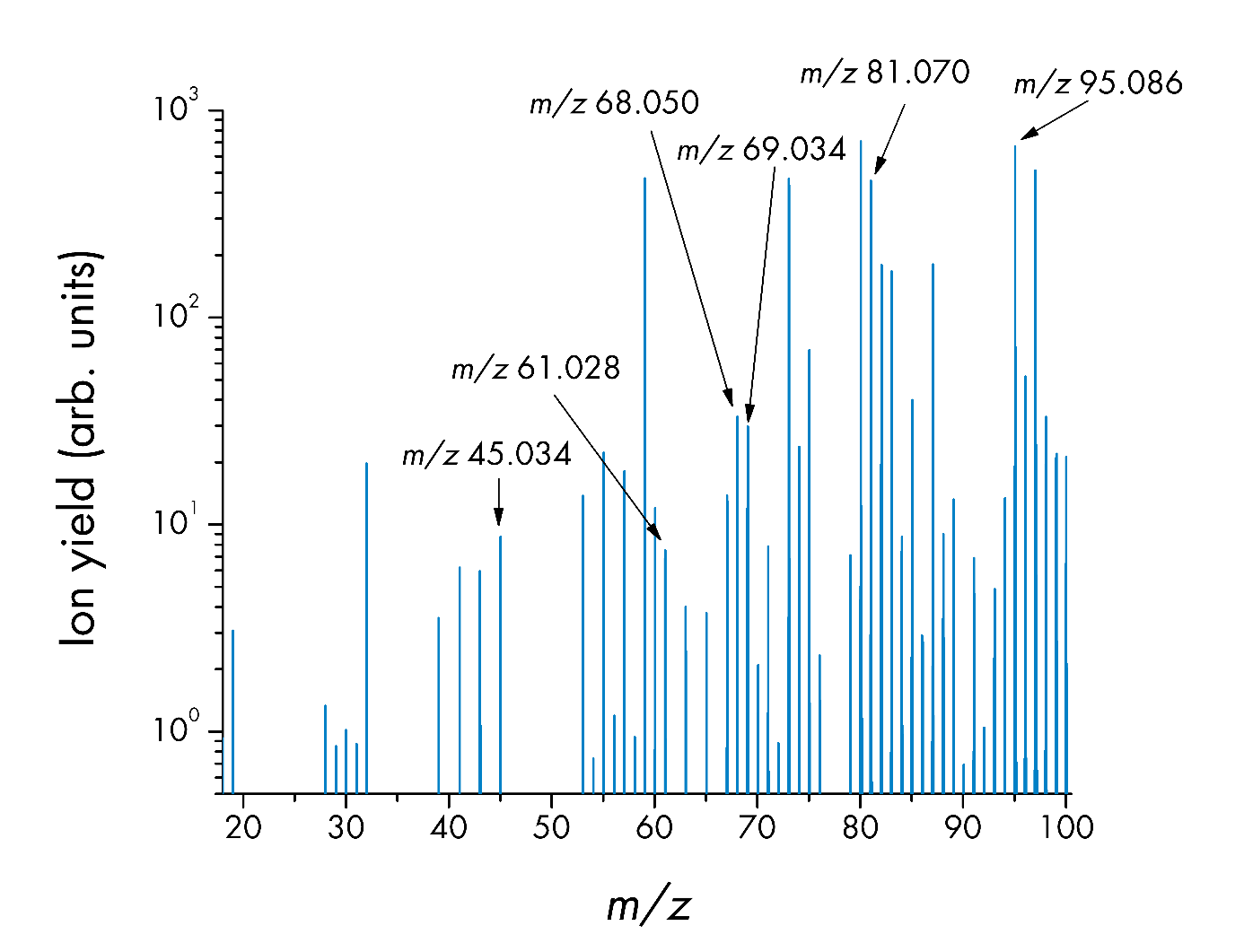

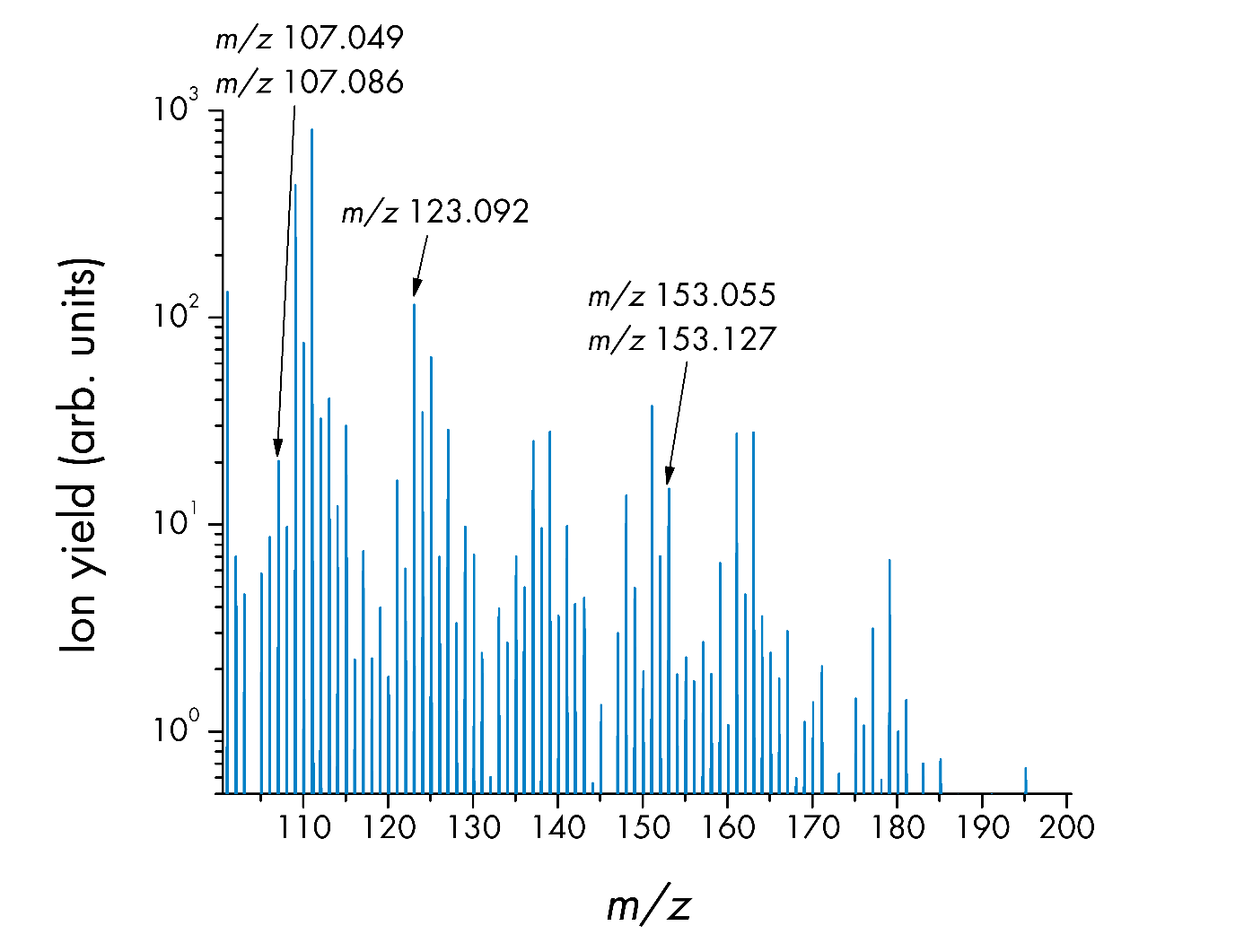


b)

Figure S4: Mass spectra sections for: a) isobaric compounds at nominal *m/z* 107, b) system from Fig. 6 at nominal *m/z* 123, c) isobaric compounds at nominal *m/z* 153. Fitted peaks are labeled with their exact *m/z* and a tentative identification.

b)


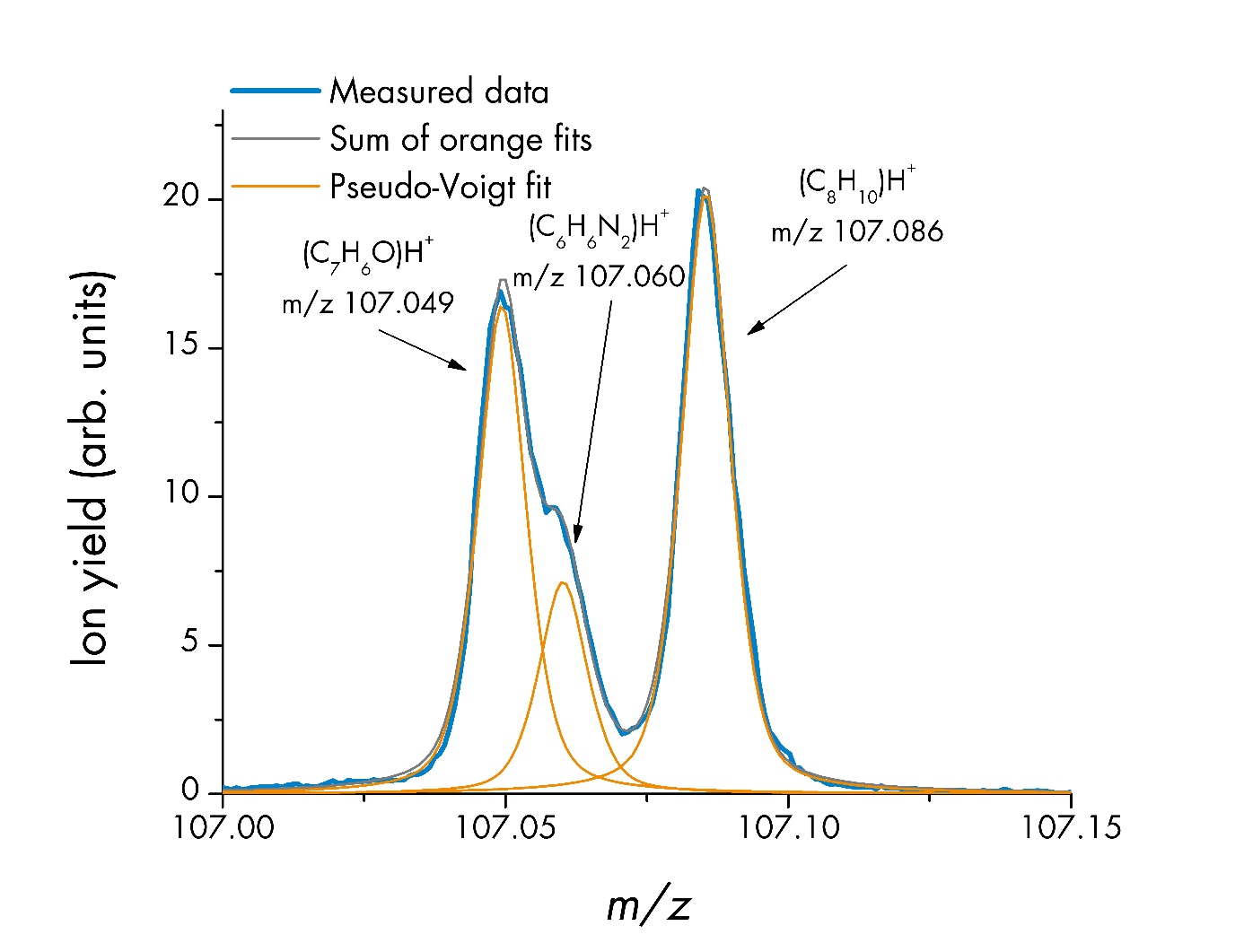

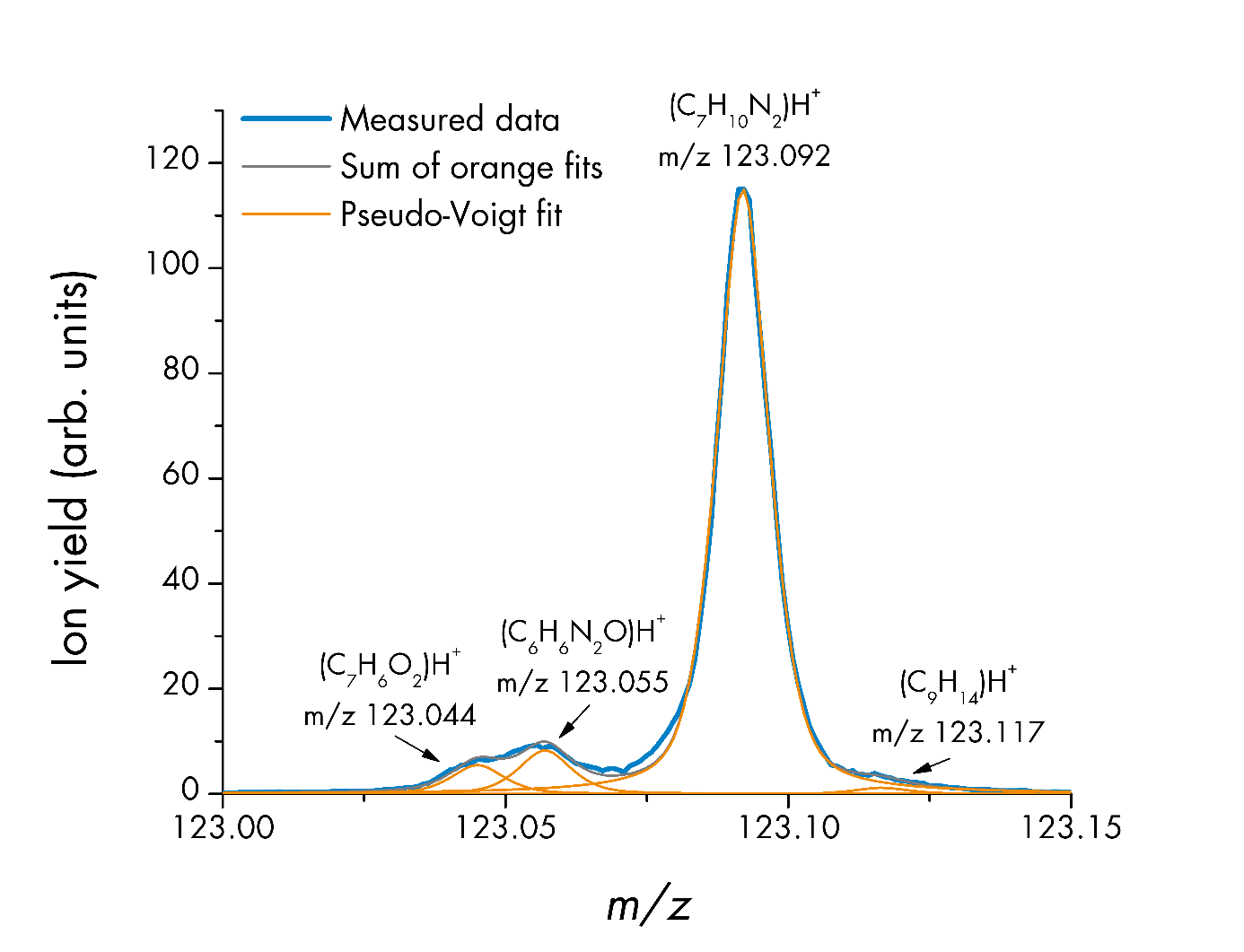


a)


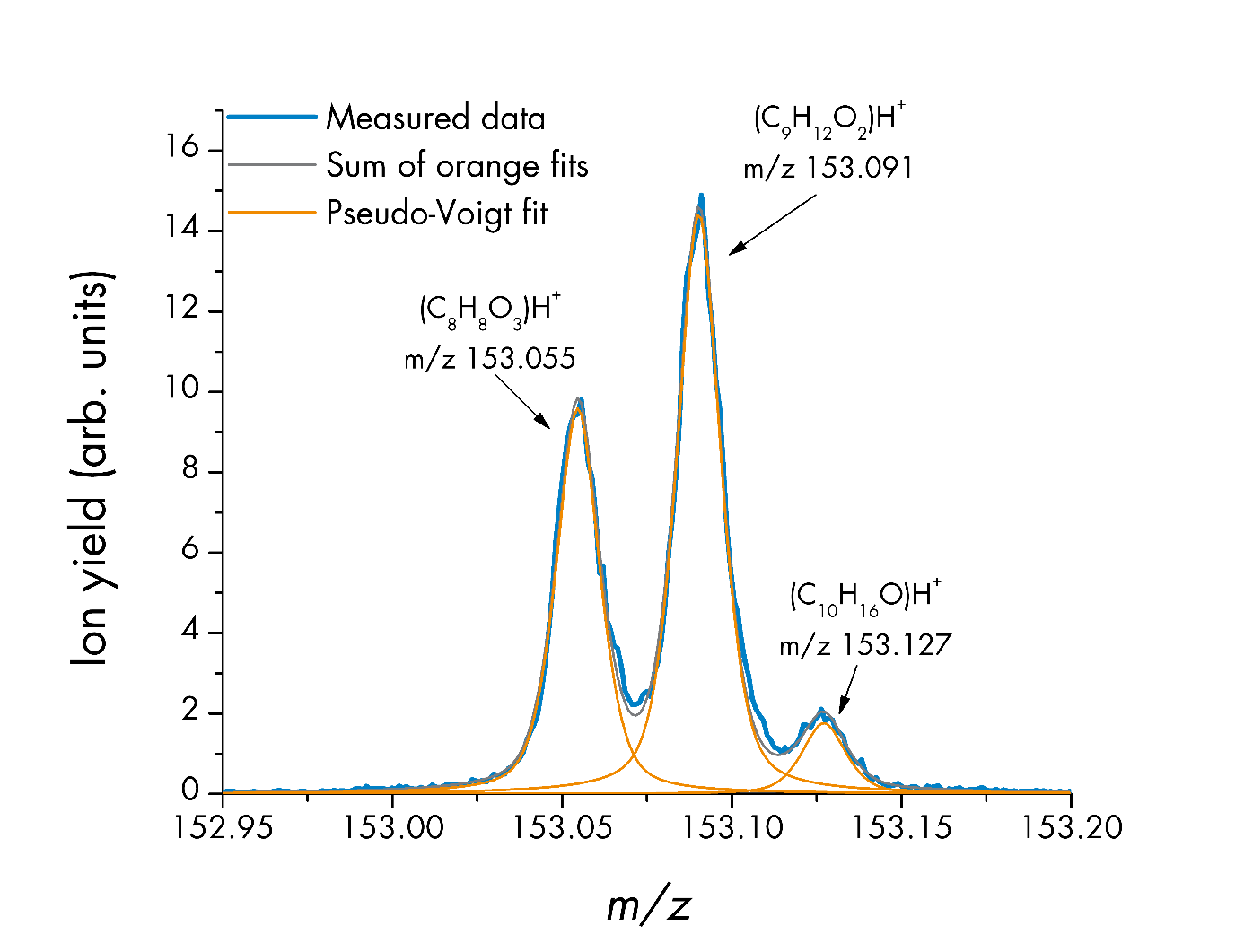


c)

Figure S5: Coffee headspace IMS spectra for selected *m/z*. Upper graph = linear, lower graph = log y-axis.


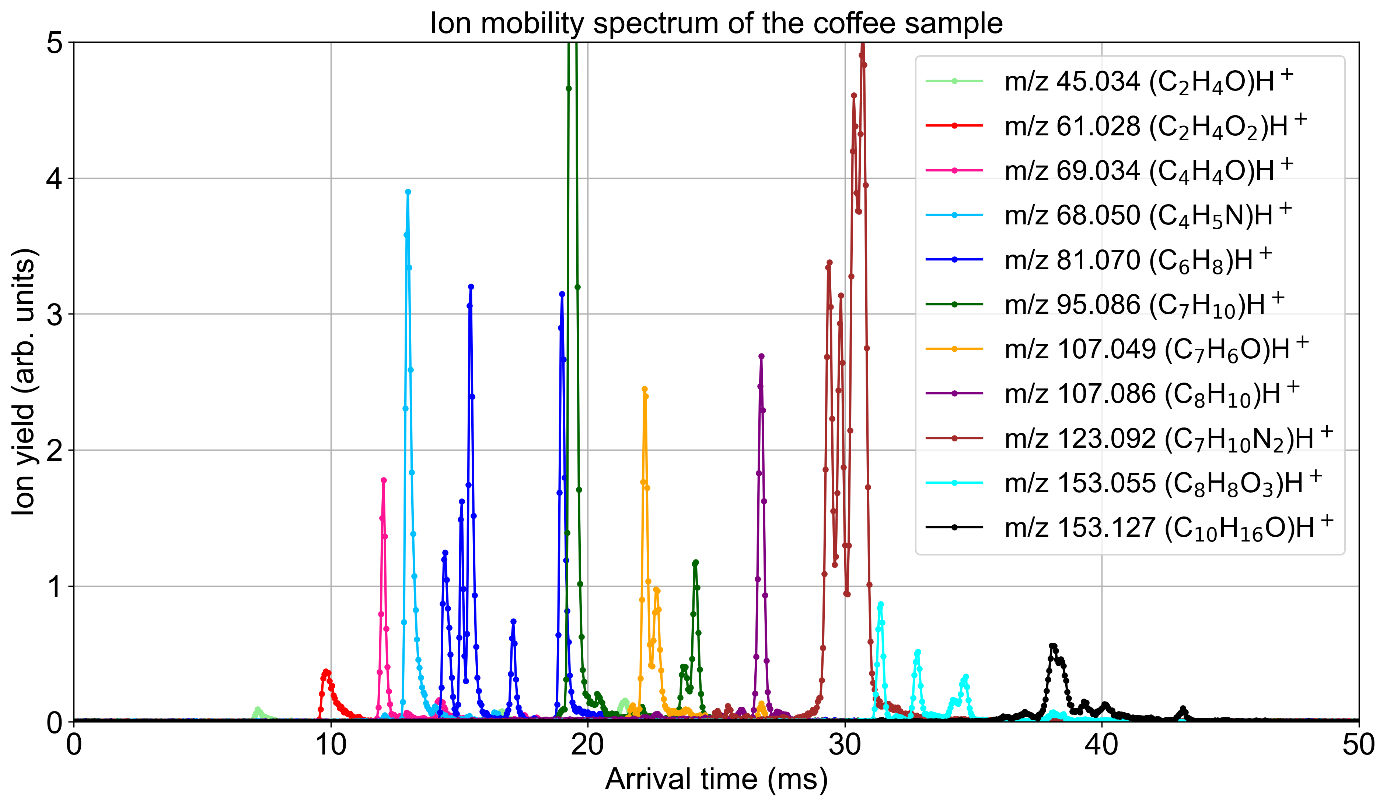


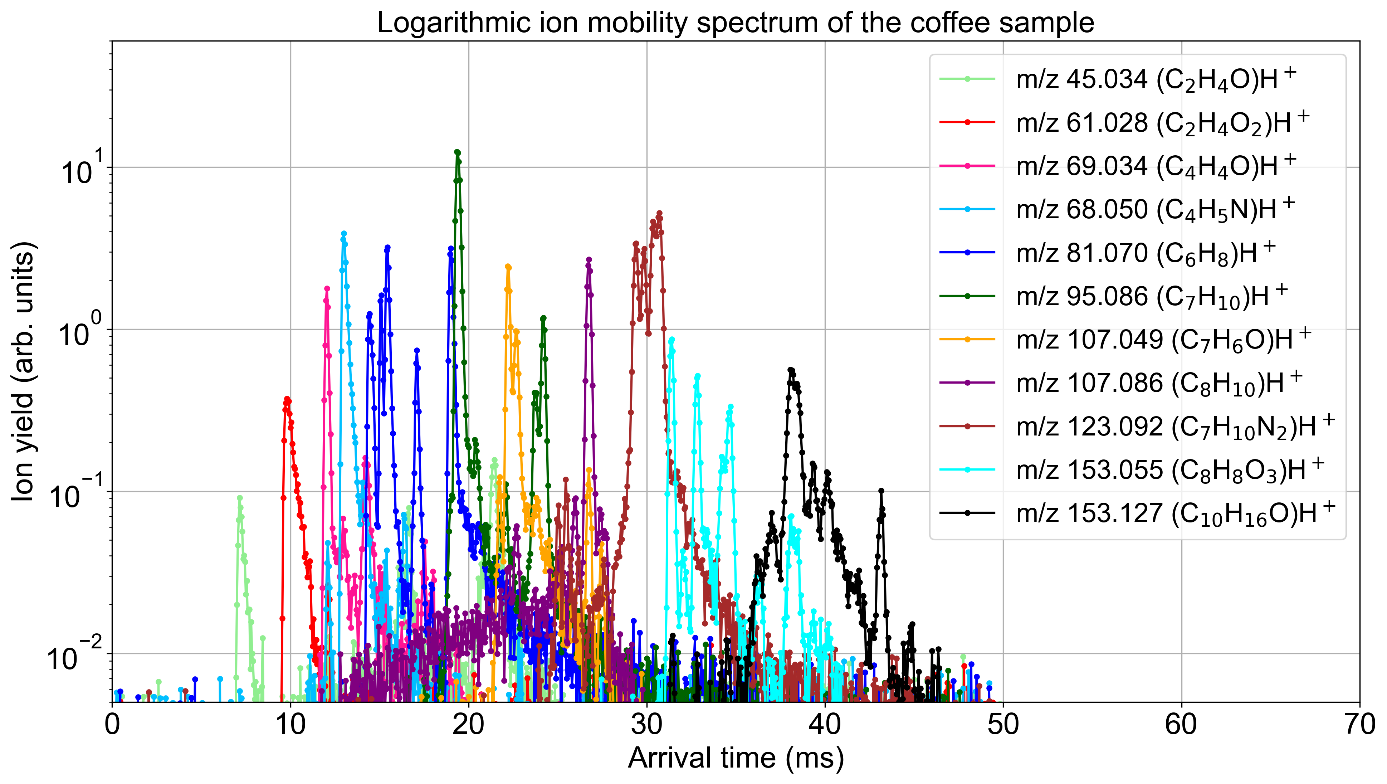


References

1. M.O. Gnioua, P. Spanel, A. Spesyvyi, Gas-phase ion mobility of protonated aldehydes in helium

   measured using a selected ion flow-drift tube. Rapid Communications in Mass Spectrometry 38/14 (2024) e9767. DOI: 10.1002/rcm.9767 [↑](#endnote-ref-1)
